# Factors influencing the missed nursing care in patients from a private hospital

**DOI:** 10.1590/1518-8345.1227.2877

**Published:** 2017-07-10

**Authors:** Raúl Hernández-Cruz, María Guadalupe Moreno-Monsiváis, Sofía Cheverría-Rivera, Aracely Díaz-Oviedo

**Affiliations:** 1 MSc, Professor, Facultad de Enfermería, Universidad Autónoma de San Luis Potosí, San Luis Potosí, Mexico.; 2 PhD, Researcher, Facultad de Enfermería, Universidad Autónoma de Nuevo León, Nuevo León, Mexico.; 3 MSc, Researcher, Facultad de Enfermería, Universidad Autónoma de San Luis Potosí, San Luis Potosí, México.; 4 PhD, Researcher, Facultad de Enfermería, Universidad Autónoma de San Luis Potosí, San Luis Potosí, México.

**Keywords:** Nursing Care, Patient Care, Human Resources, Communication, Hospitals, Private

## Abstract

**Objective:**

to determine the factors that influence the missed nursing care in hospitalized patients.

**Methods:**

descriptive correlational study developed at a private hospital in Mexico. To identify the missed nursing care and related factors, the MISSCARE survey was used, which measures the care missed and associated factors. The care missed and the factors were grouped in global and dimension rates. For the analysis, descriptive statistics, Spearman’s correlation and simple linear regression were used. Approval for the study was obtained from the ethics committee.

**Results:**

the participants were 71 nurses from emergency, intensive care and inpatient services. The global missed care index corresponded to M=7.45 (SD=10.74); the highest missed care index was found in the dimension basic care interventions (M=13.02, SD=17.60). The main factor contributing to the care missed was human resources (M=56.13, SD=21.38). The factors related to the care missed were human resources (r_s_=0.408, p<0.001) and communication (r_s_=0.418, p<0.001).

**Conclusions:**

the nursing care missed is mainly due to the human resource factor; these study findings will permit the strengthening of nursing care continuity.

## Introduction

In recent years, the health service quality in Mexico has figured on the permanent agenda of the National Health System, in response to the adverse events in health that entail unfavorable effects for the care quality and safety. Health care safety is a process that centers on the knowledge about the risks of adverse events, the elimination of unnecessary risks and the prevention of evitable events through evidence-based interventions whose efficacy has been proven. Errors and care incidents generally precede the adverse events, as well as omissions in care. When these omissions correspond to nursing care, they are called nursing care missed, leading to a large number of adverse events in the hospital context^1^.

Patient safety, considered as the prevention of patient damage, requires solid systems that prevent the errors; if they occur, they serve as a source of learning, generating a safety culture that involves all health professionals, organizations and the patients themselves. Although all health professionals play a relevant role in patient safety, nursing has a fundamental role due to its involvement in most hospital processes, making it the professional category that stands closest to the patients and a key agent to reduce adverse outcomes^2^.

The identification of the care missed permits the provision of useful information to the nursing service management with care quality and safety. For the sake of this study, the medium-range theory called the Model of Nursing Care Missed^3^ was used as the theoretical framework, which recovers three concepts from Donabedian’s Quality Model from 1966, structure, process and outcome. The Model of Nursing Care Missed argues that the structure refers to the characteristics of the hospital, the patient care service and the nursing staff. The factors related to the human resources available to deliver care, interdisciplinary team communication and available material resources needed to develop the patient care activities are also included^3^ ^-^ ^4^. The process refers to the care the nursing staff provides; when this does not take place in accordance with the patients’ needs, this is called nursing care missed, which refers to any aspect of care required by the patient that is omitted or significantly delayed. This is related with human resources, communication and material resource factors. The outcome refers to the direct effects of care on the patients. The presence of nursing care missed can cause negative outcomes, such as dissatisfaction, falls, pressure ulcers, infections, among others, all of which entail repercussions for the quality and safety of care^3^.

The authors of the Model mention that the nursing care is incorporated in four dimensions: individual needs, discharge planning and education, basic care and care with continuing assessments^5^. In the interventions in individual needs, the nursing staff uses its knowledge and skills to treat the people’s human reactions rather than the health problems and uses the person’s willingness to promote self-care and offer emotional support^6^ ^-^ ^7^. The discharge planning and education help the patient and family to improve their participation and to make informed decisions about the care; the education includes both the knowledge needed during the care process and when the patient is discharged^5^. The basic care interventions are actions to satisfy the patients’ basic needs and lack of autonomy, as the user cannot do so by himself; this is considered routine nursing care at most hospitals^8^. The care interventions with continuing assessments are involved in the continuous surveillance process and the continuing assessment of the care provided, aiming to identify any change in the patient’s health status and to make decisions on the care process^9^ ^-^ ^10^.

The nursing staff is responsible for the care quality provided. Therefore, identifying care omissions and factors related to these omissions permits taking the relevant measures involved in the restructuring of nursing services, in order to contribute to the solution of the missed nursing care problem^5^, which enhances the quality and safety of patient care.

Therefore, the objective was to determine the factors influencing the nursing care missed in hospitalized patients.

## Method

A descriptive and correlational study was undertaken at a private hospital in the State of San Luis Potosí, Mexico between January and March 2015. The complete nursing staff at the Emergency, Intensive Care and Inpatient Services was included, so that 71 nurses participated.

To collect the information, the MISSCARE^11^ survey was used, an instrument that measures the care missed and associated factors. It consists of 41 assertions, divided in three parts. The first part contains information on the sociodemographic data, providing information on the participants’ professional data. The second part, which the authors call part A, consists of 24 assertions related to the care omitted, grouped in four dimensions: interventions in individual needs, discharge planning and patient education, basic care interventions and care interventions with continuing assessments. The response range consists of a Likert scale with the following answers: 0 does not apply, 1 never, 2 rarely, 3 sometimes, 4 frequently and 5 always missed. The option “does not apply” was included for all questions of nursing care that is not provided during the night shift, such as patient meals, walking, among others. According to the authors, the response alternatives are transformed into a dichotomous scale, in which the alternatives 1, 2 and 3 are considered as care provided, while alternatives 4 and 5 are considered as care missed. The reliability coefficient of this part was 0.91. The third part, which the authors call part B, addresses the factors associated with the care missed, consisting of 17 assertions, which are grouped into human resources, communication and material resources. It contains a Likert scale with the following alternatives: 1 no reason, 2 lesser reason, 3 moderate reason and 4 significant reason; the reliability coefficient for this part corresponded to 0.90. The authors authorized the use of the instrument^11^. The original version of the instrument is in English. Therefore, the survey had to be translated from English to Spanish by two certified agencies for its semantic validation and the achievement of a consensus version in Spanish, with the support of qualified translators. Next, a pilot test was undertaken to verify the semantic clarity of the assertions^12^.

To collect the data, the services were visited during each of the different shifts and the nurses were invited to participate in the study; therefore, they received explanations about the objective and those who accepted to participate received a yellow envelope with the MISSCARE survey. It was verified that this would not interfere in their work activities and, finally, they received instructions for completion.

It should be highlighted that the study complied with the determinations of the *Norma Oficial Mexicana en materia de Investigación* ^13^. Approval for the study was obtained from the Research Ethics Committee at the Faculty of Nursing of *Universidad Autónoma de San Luis Potosí*, Mexico under registration CEIFE-2014-101. All participants were asked to sign the Informed Consent Form and the respect for their dignity, privacy, wellbeing and rights was guaranteed throughout the data collection.

To process the data, the statistical software SPSS (Statistical Package for the Social Sciences, version 20) was used to design a global index of care missed, as well as for each of the dimensions, ranging from 0 to 100, in which a higher index corresponds to a higher level of care missed. In addition, indices were designed for the factors related to the care lost, in which higher scores corresponded to higher degrees of importance for the nurses.

The Kolmogorov-Smirnov test was used to determine the normality of the data. The continuous variables did not show normal distribution, so that non-parametric tests were used, such as Kruskal-Wallis and Mann-Whitney’s U-test to identify differences in the care lost according to the assigned service, the nurses’ category, level of education, experience at the service, professional experience and shift.

To determine the influence of the human resources, communication and material resources on the nursing care missed, first, Spearman’s correlation analysis was applied among the coefficients of each factor, the intervention dimension coefficients and the global coefficient. Then, simple linear regression was applied to determine the effect of the factors on the care lost.

## Results

Female nurses were predominant in the study with 77.5%, with a mean age of 28.4 years (SD=5.61). The predominant age group was between 26 and 30 years old (45.1%), followed by 21 to 25 years (35.2%). Ninety-three percent (93.0%) of the staff corresponded to baccalaureate nurses, while the other professionals were auxiliary nurses.

Seventy-one point eight percent held a Teaching Diploma in Nursing; 35.2% had worked at the institution between three and four years and 47.9% had worked at the service between one and two years. Concerning the professional experience, 62% possessed between one and five years and the night shift was the predominant work shift (42.2%), followed by the morning shift (33.8%). The mean number of patients assigned per nurse was six (SD=4), with three incoming and departing patients per shift.

### Elements of the missed nursing care

In Table 1, the global missed nursing care index and the dimension indices are displayed; the main care omission corresponds to the basic care interventions, and the dimension with the lowest care omission was interventions with continuing assessments. The average global care index was 7.45 (SD=10.74).

**Table 1:** Global and dimension indices of nursing care missed at a Private Hospital in the State of San Luis Potosí, Mexico, 2015

**Indices**	**Mean**	**Median**	**Standard deviation**	**95% confidence interval**	
				**Lower limit**	**Upper limit**
**Interventions in individual needs**	**5.03**	**0.0**	**9.06**	**2.88**	**7.17**
**Discharge planning and patient education**	**5.63**	**0.0**	**18.02**	**8.86**	**17.19**
**Basic care interventions**	**13.02**	**0.0**	**17.60**	**1.36**	**9.90**
**Care interventions with continuing assessments**	**4.02**	**0.0**	**13.9**	**0.73**	**7.31**
**Global missed nursing care index**	**7.45**	**4.16**	**10.74**	**4.90**	**9.99**

Source: MISSCARE survey for Nursing staff

Concerning the interventions for basic care, the most missed or omitted care element is mouth care (28.2%), followed by help with walking three times per day or as indicated and patient feeding when the food is still warm (both 19.7%); the least omitted care was foot and wound care (1.4%).

In the discharge planning and patient education dimension, the most missed care was patient teaching while in hospital (7%) and the least omitted care the planning of patient discharge and teaching (4.2%).

In the individual needs dimension, the most missed care element is the emotional support to the patient and/or family (14.1%), followed by help with interdisciplinary care assessment visits (8.5%). No missed care was identified in the assessment of drug efficacy (0%).

Regarding the dimension of care interventions with continuous assessments, the most missed care was the complete patient documentation with the necessary data and the performance of the patient evaluations per shift (each 5.6%); the least omitted care, however, corresponded to the fluid balance-control of incoming and departing patients (1.4%).

Regarding the differences in care missed according to the nurses’ labor characteristics, the only difference was found in the assigned service (X2 = 5.82, p = 0.05). The care missed was more predominant at the inpatient service when compared to the emergency department (U = 166.5, p = 0.045).

### Factors influencing the missed care

In Table 2, the indices of the human resource, communication and material resource factors are displayed that influence the care missed. The nurses signaled that the main factor influencing the care missed relates to nursing human resources, with an average index of 56.13 (SD=21.38), followed by communication and, finally, material resources.

**Table 2:** Dimension indices of missed nursing care the nursing care missed at a Private Hospital in the State of San Luis Potosí, Mexico, 2015

**Indices**	**Mean**	**Median**	**Standard deviation**	**95% confidence interval**	
				**Lower limit**	**Upper limit**
**Human resources**	**56.13**	**57.14**	**21.38**	**51.07**	**61.19**
**Communication**	**48.55**	**47.61**	**23.42**	**43.01**	**54.10**
**Material resources**	**45.07**	**44.44**	**29.80**	**38.01**	**52.12**

Source: MISSCARE survey for Nursing staff

Concerning the human resource factor, the nurses mentioned that the elements in which they receive a significant reason for missed nursing care superior to 40% correspond to insufficient staff, followed by the unexpected increase in the patient volume and/or the work burden at the service (39.4%).

With regard to the communication elements, the nurses perceive the nurse’s unavailability when the patient calls on her (22.5%) as a significant reason, followed by tension or errors in the communication with the medical staff (21.1%).

As for the elements that correspond to the material resource factor, the nurses signaled the unavailability of the drugs when necessary (21.1%) as a significant reason, followed by the supplies and equipment (16.9%).

### Factors influencing the dimensions of nursing care missed

To prove the influence of the missed nursing care factors, first, Spearman’s correlation coefficient was applied. A significant and positive relation was found between the human resources and the individual needs, basic care, continuing assessment interventions and the global index. The communication factors were associated with the individual needs and basic care interventions and with the global index. It should be highlighted that the factors related with the material resources were not associated with any of the care dimensions (Table 3).

**Table 3:** Spearman correlation coefficients of care dimensions and factors contributing to omissions of nurses at a Private Hospital in the State of San Luis Potosí, Mexico, 2015

**Care dimensions**	**Factors for missed care**		
	**Human resources**	**Communication**	**Material resources**
**Interventions in individual needs**	0.327*	0.324*	**-0.080**
**Discharge planning and patient education**	**0.110**	**0.103**	**-0.038**
**Basic care interventions**	0.349*	**0.391** ^**†**^	**-0.175**
**Care interventions with continuing assessments**	0.282*	**0.211**	**-0.149**
**Global missed nursing care index**	**0.408** ^**†**^	**0.418** ^**†**^	**-0.193**

*p<0.01; †p<0.001

Source: MISSCARE survey for Nursing staff

After determining the relation between the variables, simple linear regression analysis was applied to determine the influence of human and communication factors in care missed. The human resources explain 13% of the global care lost and communication 14% (Table 4).

**Table 4:** Nurses’ perception of factors affecting the care provided to inpatients at a Private Hospital in the State of San Luis Potosí, Mexico, 2015

**Determinants**	**βeta**	**Standardized βeta**	**t**	**p**	**R** ^**2**^
**Human resources**	**0.18**	**0.37**	**3.35**	**0.001**	0.13*
**Communication**	**0.17**	**0.38**	**3.5**	**0.001**	0.14*

*p<0.001

Source: MISSCARE survey for Nursing staff

## Discussion

Based on the study results, it could be identified that there are elements of nursing care that are missed or omitted during the patients’ hospital stay, as little more than half of the nurses indicated that at least one care is lost during the patient’s hospital stay.

The care dimension where nurses perceive the greatest omission is that of basic care interventions, followed by discharge planning and patient education interventions. As for the dimension of basic care interventions, it is similar to a study from 2009^5^, but it differs in its proportion, which was lower in the present study, whereas the previously reported study found a much higher proportion (73%). The execution of basic nursing interventions is essential during the patients’ hospital stay, especially in patients who lack autonomy due to their health condition^14^. The omission in this type of interventions can be attributed to the fact that nurses do not prioritize them, either because of the low complexity of their condition or because they consider that the patient can perform these care actions by himself or with the support of a relative^15^.

Regarding the elements in the dimension of basic care interventions, the nurses reported greater omission in mouth care and help with walking three times a day or as indicated; these results are similar in proportion to a study carried out in 2011^4^ and another in 2006^15^, but they differ from other studies^5^ ^,^ ^16^, where the proportion was much higher. The importance of knowing these omissions is that a significant association exists between missed care and adverse events. Care omissions while walking have been associated with falls in hospitalized patients^17^.

In the planning dimension of patient discharge and education, nurses pointed to a lack of patient education about the disease, tests and diagnostic studies. This result is similar to the findings in some studies^5^ ^,^ ^15^ ^,^ ^18^, but differs in proportion, as those studies have reported greater omission. This can be attributed to the characteristics of the institutions where the studies were carried out. According to the literature ^3^ ^,^ ^15^, these aspects are important, as the lack of education prior to hospital discharge has a negative impact on hospital outcomes, such as complications and hospital readmissions.

As for the interventions in individual needs, the nurses perceived fewer omissions. The reported omission is relevant to consider though, especially since care actions are intended to respond to human needs rather than to health problems^6^ ^-^ ^7^. The most missed care element according to the nurses was emotional support to the patient, with similar results, but to a lesser extent than in some previous studies^17^ ^-^ ^18^. Some authors^4^ ^,^ ^15^ ^,^ ^19^ point out that the omission can be attributed to the time required for their execution, which nursing often allocates to other care that they consider a priority, such as physician-delegated interventions.

Another element of this dimension the staff perceived with greater omission was help with the interdisciplinary assessment visits. The omission in this intervention is similar, although to a lesser extent than in a study from 2011^4^. This could be due to the high work demands as well as to the work system, where interdisciplinary teamwork often is not designed^15^.

Finally, in the dimension of care interventions with continuing evaluations, although the staff perceived little care missed, there are elements that are omitted, such as the complete patient documentation with the necessary data and patient evaluations per shift. These results are similar to some studies^5^ ^,^ ^15^ ^,^ ^17^, but to a lesser extent than reported in those studies.

Some authors^4^ ^-^ ^5^ ^,^ ^18^ point out that missed care can vary according to some characteristics of the nurses, such as the appointed service, level of education, assigned category, length of experience at the institution and service, work experience and work shift. In this study, however, the only difference found was related to the appointed service. This could be attributed to the organizational characteristics of the institutions where the studies were carried out, where the profile and role of the nurse they play may be different from the Mexican context. The difference observed corresponded to the emergency department and inpatient service with greater omission at the latter. This can be attributed to the difference of activities between the services studied.

Another important finding is the factors that are attributed to missed care; in the present study, the staff considered that the main factor corresponds to nursing human resources, followed by communication and finally material resources. Regarding the relevance of human resources for the omission of care, the findings are consistent with previous studies^(4-5, 20)^, although they report a greater proportion. For the present study, we also looked for an association between missed care and these factors, finding no association between missed care and its dimensions for the material resources. This finding could be attributed to the fact that the present study was carried out at a private institution, where material resources are generally available to cover the care demands.

Nurses consider human resources as the main factor for missed care, where they mentioned that the insufficient number of staff and the unexpected increase in the volume of patients and / or service workload are the most significant elements, which is similar, but to a lesser extent than the findings in 2011^4^ and 2009^5^. At services with limited human resources, nurses decrease or sometimes omit interventions, although this may increase the risk of negative outcomes in the patient^21^ ^-23^. A relevant finding in the present study was that the HR factor mainly affects interventions in individual needs and basic care interventions.

In the communication factor, nurses perceive the unavailability of the nurse responsible for the patient when the patient requests it as an important reason, followed by tension or errors in communication with the medical staff. The latter finding is similar to that reported in 2011^4^, although the authors mention greater proportions. The literature^20^ mentions that interdisciplinary communication favors the continuity of care, besides avoiding errors in health care. Therefore, health institutions should strengthen interdisciplinary work. In this sense, the nursing managers could modify the work system at the organizational level. It should be noted that, in addition to the fundamental importance of communication with the interdisciplinary team, in the present study, it was found that communication affects the execution of basic care interventions and interventions in individual needs. Nursing managers should take this finding into account to implement strategies that allow for effective communication among all those involved in patient care.

Regarding the elements of the material resource factor, nurses mentioned the unavailability of medicines when needed as an important reason. This result is in line with that reported in 2011^4^ and 2009^5^, although those authors report a greater proportion. The previously mentioned studies indicate that the availability of drugs avoids unnecessary delays in the pharmacological treatment of patients, thus contributing to the continuity of care.

Finally, it is important to highlight that the findings will allow nursing managers to make decisions aimed at strengthening the continuity of care. Nevertheless, the patients’ opinions should be considered, an aspect not included in the present study and that can constitute a limitation. Patients’ opinions, as the main recipients of nursing care, permit greater clarity in the phenomenon of missed care, thus generating effective strategies that contribute to improve the quality and safety of care.

## Conclusion

Missed nursing care represents omissions in care for patients during their hospital stay. The findings of the present study reveal that the interventions for basic care and the planning of discharge and patient education are the interventions in which there is greater omission. Both represent independent nursing care that should not be missed or omitted during the patients’ stay.

Missed care was associated with factors related to human resources and communication. It should be noted that no association was found between missed care and material resources. Human factors are a key aspect that is directly related to the results of patient care. Therefore, the nursing managers need to manage and have competent and sufficient nursing staff to satisfy the care demands, as well as to strengthen effective communication between nursing professionals and the rest of the clinical staff involved in care, in order to strengthen nursing care and contribute to the quality and safety of hospital care.
